# Metastatic Uveal Melanoma Surveillance: A Delphi Panel Consensus

**DOI:** 10.3390/cancers18010121

**Published:** 2025-12-30

**Authors:** Juan Alban, R. Christopher Bowen, David A. Reichstein, Meredith McKean, Jose Lutzky, Ezekiel Weis, Richard D. Carvajal, Susan Dulka, Brian G. Morse, Marcus O. Butler, Suthee Rapisuwon, Kevin B. Kim, Sanjay Chandrasekaran, Allison Betof Warner, Jonathan S. Zager, Bartosz Chmielowski, Sapna P. Patel, Leonel Fernando Hernandez-Aya, Zelia M. Correa, Leslie A. Fecher, Yana G. Najjar, Kamaneh Montazeri, Alexander N. Shoushtari, Asad Javed, Dan S. Gombos, April K. S. Salama, Katy Tsai, Frank H. Miller, Nikhil Khushalani, Rino S. Seedor, Evan J. Lipson, Sunil A. Reddy, Elizabeth Buchbinder, Shailender Bhatia, Anna Pavlick, Inderjit Mehmi, Thomas Aaberg, Alexandra P. Ikeguchi, Ivana K. Kim, Scott D. Walter, Arun D. Singh, Ryan J. Sullivan, Jacob S. Choi, Basil K. Williams Jr., Marlana Orloff, Prithvi Mruthyunjaya, Megan D. Schollenberger, Namita Gandhi, J. William Harbour, Sunandana Chandra

**Affiliations:** 1 Robert H. Lurie Comprehensive Cancer Center, Division of Hematology Oncology, Northwestern University, Chicago, IL 60616, USA; 2 Department of Ophthalmology, Northwestern University, Chicago, IL 60616, USA; 3 Tennessee Retina, Nashville, TN 37203, USA; 4 Sarah Cannon Research Institute, Nashville, TN 37203, USA; 5 Sylvester Comprehensive Cancer Center, University of Miami, Miami, FL 33136, USA; 6 Department of Ophthalmology, Faculty of Medicine and Dentistry, University of Alberta, Edmonton, AB T5H 3V9, Canada; 7 Northwell Health Cancer Institute, New York, NY 11042, USA; 8 Department of Radiology, Thomas Jefferson University, Philadelphia, PA 19107, USA; 9 Department of Diagnostic Imaging and Interventional Radiology, Moffitt Cancer Center, Tampa, FL 33612, USA; 10 Princess Margaret Cancer Centre, University Health Network, Departments of Medicine and Immunology, University of Toronto, Toronto, ON M5G 2M9, Canada; 11 Department of Oncology, Lombardi Comprehensive Cancer Center, Georgetown University Medical Center, Washington, DC 20007, USA; 12 Center for Melanoma Research and Treatment, California Pacific Medical Center Research Institute, San Francisco, CA 94115, USA; 13 Division of Hematology/Oncology, Department of Internal Medicine, Harold C. Simmons Comprehensive Cancer Center, University of Texas Southwestern Medical Center, Dallas, TX 75235, USA; 14 Division of Oncology, Department of Medicine, Stanford University School of Medicine, Stanford, CA 94305, USA; 15 Departments of Cutaneous Oncology, Moffitt Cancer Center, Tampa, FL 33612, USA; 16 Division of Hematology and Medical Oncology, Department of Medicine, Jonsson Comprehensive Cancer Center, The University of California Los Angeles, Los Angeles, CA 90095, USA; 17 Division of Medical Oncology, Department of Medicine, University of Colorado, Aurora, CO 80045, USA; 18 Department of Ophthalmology, University of Colorado, Aurora, CO 80045, USA; 19 Division of Hematology and Oncology, Department of Internal Medicine, University of Michigan Rogel Cancer Center, Ann Arbor, MI 48109, USA; 20 UPMC Hillman Cancer Center, Pittsburgh, PA 15232, USA; 21 Massachusetts General Cancer Center, Harvard Medical School, Boston, MA 02115, USA; 22 Department of Medicine, Memorial Sloan Kettering Cancer Center, New York, NY 10065, USA; 23 Weill Cornell Medical College, New York, NY 10065, USA; 24 Holden Comprehensive Cancer Center, University of Iowa, Iowa City, IA 52242, USA; 25 MD Anderson Cancer Center, Section of Ophthalmology, Department of Head and Neck Surgery, The University of Texas, Houston, TX 77030, USA; 26 Division of Medical Oncology, Duke University, Durham, NC 27710, USA; 27 Department of Medicine, Division of Hematology/Oncology, University of California, San Francisco (UCSF), San Francisco, CA 94143, USA; 28 Department of Radiology, Northwestern University Medical Center, Chicago, IL 60601, USA; 29 Sidney Kimmel Comprehensive Cancer Center, Thomas Jefferson University, Philadelphia, PA 19107, USA; 30 Department of Oncology, Sidney Kimmel Comprehensive Cancer Center, Johns Hopkins University, Baltimore, MD 21287, USA; 31 Department of Medical Oncology, Dana-Farber Cancer Institute, Boston, MA 02215, USA; 32 Division of Hematology and Oncology, University of Washington, Fred Hutchinson Cancer Center, Seattle, WA 98109, USA; 33 Department of Medical Oncology, Weill Cornell Medicine, New York, NY 10021, USA; 34 The Angeles Clinic and Research Institute, Cedars Sinai Affiliate, Los Angeles, CA 90025, USA; 35 Retina Specialists of Michigan, Foundation for Vision Research, Michigan State University College of Human Medicine, Grand Rapids, MI 49546, USA; 36 Department of Melanoma Medical Oncology, University of Texas MD Anderson Cancer Center, Houston, TX 73030, USA; 37 Massachusetts Eye and Ear and Department of Ophthalmology, Harvard Medical School, Boston, MA 02114, USA; 38 Retina Consultants, Hartford, CT 06106, USA; 39 Department of Ophthalmic Oncology, Cole Eye Institute, Cleveland Clinic, Cleveland, OH 44106, USA; 40 Byers Eye Institute, Stanford University School of Medicine, Watson Court, Palo Alto, CA 94303, USA; 41 Department of Radiology, Cleveland Clinic, Cleveland, OH 44195, USA; 42 Department of Ophthalmology, Simmons Comprehensive Cancer Center, University of Texas Southwestern Medical Center, Dallas, TX 75390, USA

**Keywords:** uveal melanoma, PRAME, gene-expression profile, metastatic uveal melanoma surveillance

## Abstract

Background/Objectives: Uveal melanoma is a rare but aggressive intraocular malignancy that metastasizes in up to half of patients, most commonly to the liver, despite effective local treatment. In the absence of robust evidence, there are no standardized guidelines for post-treatment surveillance, resulting in wide variation in imaging modalities, frequency, and duration across physicians and institutions. This study aimed to develop expert consensus recommendations for surveillance strategies in patients with uveal melanoma. Methods: A modified Delphi method was conducted across three iterative survey rounds between September 2024 and February 2025 using an online platform. Panelists included medical oncologists, ocular oncologists, radiologists, and surgical oncologists from North America. A multidisciplinary steering committee developed statements addressing risk-based surveillance using both molecular and clinical prognostic factors, including gene expression profiling (GEP) and PRAME status. Consensus was defined a priori as ≥70% of panelists rating a statement 7–9 on a 9-point Likert scale. Results: Forty-nine experts were invited, and 41 completed at least one survey round. The panel represented 17 U.S. states, Washington, D.C., and two Canadian provinces. Twelve statements reached stable consensus, including recommendations for imaging modality, frequency, and duration in intermediate- and high-risk patients. Although there was agreement that low-risk patients warrant surveillance, no consensus was reached on the optimal approach for this group. Conclusions: This is the first study to provide consensus-based guidance incorporating GEP and PRAME status into surveillance recommendations for uveal melanoma, offering a standardized framework to guide clinical practice and future research.

## Simple Summary

Uveal melanoma is a rare but aggressive eye cancer that frequently spreads to distant organs, most commonly the liver, even after effective local treatment of the primary tumor. In the absence of standardized guidelines, current surveillance practices vary widely among physicians and institutions, creating uncertainty in patient care. To address this gap, our study convened a multidisciplinary panel of ocular oncologists, medical oncologists, radiologists, and surgeons from across North America to develop expert consensus recommendations for post-treatment surveillance. Notably, these recommendations are the first to incorporate genetic and molecular characteristics of the tumor, in addition to traditional clinical features, to guide risk-based surveillance strategies. This framework offers a standardized approach to monitoring, with the potential to improve early detection of metastatic disease and to serve as a foundation for future studies aimed at validating and refining surveillance practices in uveal melanoma.

## 1. Introduction

Uveal melanoma (UM) is a rare and aggressive form of cancer originating from melanocytes within the uveal tract of the eye, which includes the choroid, ciliary body, and iris. With an annual incidence of approximately five to six cases per million in the United States, UM represents the most common primary intraocular malignancy in adults [1]. The median age at diagnosis is 60 years [2]. Despite adequate local treatment with modalities such as plaque brachytherapy, proton beam therapy, and enucleation, up to 50% of patients with uveal melanoma are at intermediate or high risk of developing metastatic disease [3,4]. Adverse prognostic factors for survival include both molecular and clinical features. High-risk molecular features include Class 2 gene expression profile (GEP), expression of PRAME (preferentially expressed antigen in melanoma), BAP1 mutations leading to loss of BAP1 function, loss of heterozygosity on chromosome 3, and gain of chromosomes 1q, 6p, or 8q [5,6,7]. While clinicopathologic risk factors such as advanced patient age, larger tumor basal diameter, ciliary body involvement, extra-scleral tumor extension, and epithelioid cell type are associated with increased metastatic risk, two prospective studies by the Collaborative Ocular Oncology Group (COOG) have demonstrated the superiority of modern molecular prognostic methods [4,5,6,7,8]. In the First Report of COOG Study No. 2, UM patients with a Class 2 GEP experienced significantly lower 5-year metastasis-free survival (MFS) compared to those with Class 1 GEP (52.1% vs. 92.3%, *p* < 0.001) [4]. The integration of GEP and PRAME expression resulted in further stratification of UM patients as high risk (Class 2/PRAME-positive, MFS 44.8%), intermediate risk (Class 2/PRAME negative, MFS 58.3%; or Class 1/PRAME-positive, MFS 80.6%), or low risk (Class 1/PRAME negative, MFS 95.6%) for metastatic disease progression [4].

To date, there is an absence of consensus and robust empirical evidence to drive surveillance decisions based on risk of metastasis in individual patients. While current NCCN guidelines provide recommendations regarding frequency and duration of surveillance for UM, they do not specify modality and are not based on empirical evidence [9]. The United Kingdom [10], Canada [11], and Scotland [12] have also published national guidelines for UM surveillance, although none have incorporated PRAME status, a cancer–testis antigen expressed in various cancers, including UM, into surveillance recommendations. PRAME expression in UM is associated with higher metastatic risk, poorer prognosis, and adverse pathological features such as larger tumor size, higher TNM stage, and frequent chromosomal alterations [13,14]. When combined with gene-expression profiles (GEP), PRAME status has been shown to provide superior prognostic accuracy compared to other clinical or histopathological factors [4]. In the absence of clear guidelines, there is significant variation in practice patterns for UM surveillance across North America. Imaging modality, surveillance frequency, duration of surveillance, and the incorporation of extra-hepatic imaging can vary significantly from institution to institution. In the absence of sufficient empirical data, we chose the Delphi method to create a consensus document to standardize our practices and surveillance recommendations, and provide expert guidance to practitioners who may encounter this cancer infrequently in clinical practice [15].

The Delphi method is a technique used to achieve consensus among a group of experts. It was originally developed in the 1950s by research sponsored by the United States Air Force under Project RAND, in which a series of questionnaires was provided [15]. It has been subsequently modified and used in health research as a method to achieve consensus on important subjects or develop new concepts, especially when there is a lack of scientific evidence to develop guidelines or best practices. This method involves multiple rounds of questionnaires, where experts anonymously provide their opinions and feedback. After each round, the experts are provided with a summary of responses and invited to a debriefing session, allowing them to discuss and revise their views. This iterative process is continued until consensus is reached or diminishing returns are observed [16,17,18]. A modified Delphi is completed electronically, which affords experts the flexibility to complete each round on their own time. As a result, the modified Delphi method served as an ideal method to generate consensus in metastatic uveal melanoma surveillance.

This consensus effort was led by the Collaborative Ocular Oncology Group (COOG), the largest North American consortium of ocular oncologists, medical oncologists, and translational researchers dedicated to improving outcomes for patients with uveal melanoma and other ocular tumors. Established in 2004, COOG includes more than 25 academic and private ocular oncology centers and has been continuously funded by the National Cancer Institute for over a decade. Through its support of multicenter clinical trials, prospective registries, and translational research, COOG has played a pivotal role in shaping evidence-based management of rare ocular malignancies, including uveal melanoma [19]. The present Delphi study represents a coordinated, multidisciplinary initiative by COOG investigators to standardize surveillance practices in metastatic uveal melanoma in the absence of high-level evidence.

## 2. Materials and Methods

### 2.1. Steering Committee and Expert Panel

A steering committee consisting of 10 experts in medical oncology, ocular oncology, and radiology was established to guide this Delphi study. Committee members were selected to ensure a diverse range of perspectives, with an emphasis on clinical experience in managing uveal melanoma and active involvement in the field. This approach aimed to create a balanced leadership group capable of facilitating robust discussions and guiding the consensus process. To ensure a robust and representative expert panel, the committee aimed to recruit a minimum of 30 participants, consistent with prior methodological research recommending an optimal panel size of 30–50 for homogeneous Delphi studies [20]. The committee identified potential panelists by leveraging existing professional networks such as the COOG. A total of 49 experts in ocular oncology, medical oncology, radiology, and surgical oncology from over 40 UM centers of excellence in the US and Canada were recruited to participate on the Dephi panel.

### 2.2. Study Design

This modified Delphi study was conducted over three iterative rounds between September 2024 and February 2025. Surveys were distributed via an online platform, *Qualtrics^TM^*. Panelists were given three weeks to complete each round. The total number of rounds was not pre-specified; however, the study was concluded after three rounds due to the expected futility of additional iterations in achieving further consensus.

The first-round survey was developed by the steering committee. Each item focused on risk-based surveillance of metastatic uveal melanoma. Risk stratification was based on established molecular and clinical prognostic factors from the *First Report of Collaborative Ocular Oncology Group Study No. 2* [4], and all statements were categorized according to three risk groups:

- Low risk: Class 1 (1A or 1B) GEP and PRAME negative; or disomy 3 without gain of 8q; or *EIF1AX* mutation; or T1 (AJCC).
- Intermediate risk: Class 1 (1A or 1B) GEP and PRAME positive; or disomy 3 with gain of 8q; or *SF3B1* mutation; or T2 and T3 (AJCC).
- High risk: Class 2 GEP and PRAME-positive (44.8% 5-year MFS); or PRAME-negative; or monosomy 3 (regardless of 8q); or *BAP1* mutation leading to loss of function; or T4 (AJCC).

In addition to textual descriptions, panelists were provided with Kaplan–Meier survival curves from the *First Report of Collaborative Ocular Oncology Group Study No. 2* to aid in decision-making [4]. Participants were asked to assess each statement using a 9-point Likert scale, where scores of 1–3 indicated disagreement, 4–6 indicated uncertainty, and 7–9 indicated agreement.

Each survey was conducted online. Round one was completed anonymously, while rounds two and three required participants to provide their names and specialties. Participants had the option to submit written feedback after rating each statement. Following each round, numerical and written responses were collected and analyzed. A summary report was then prepared and shared with all panelists, detailing participant numbers, medical specialty distributions, response distributions for each statement, basic statistical analyses (mean, median, minimum, maximum), and the percentage of panelists scoring statements 7 or above.

Panelists subsequently participated in a virtual debrief session to discuss areas of disagreement and provide additional written and verbal feedback. Statements that achieved consensus, defined as ≥70% of panelists scoring a statement 7 or higher, for the first time were presented again in the subsequent round to ensure stability of results. Statements that did not meet the consensus threshold were revised based on panelist feedback and reintroduced in the next round. Any statement that reached consensus across two rounds was included as a consensus statement and omitted from subsequent rounds. The process continued until the study reached its third round, at which point further rounds were deemed unlikely to provide additional meaningful consensus, and the study was concluded.

## 3. Results

### 3.1. Delphi Panelists

**Figure 1:**
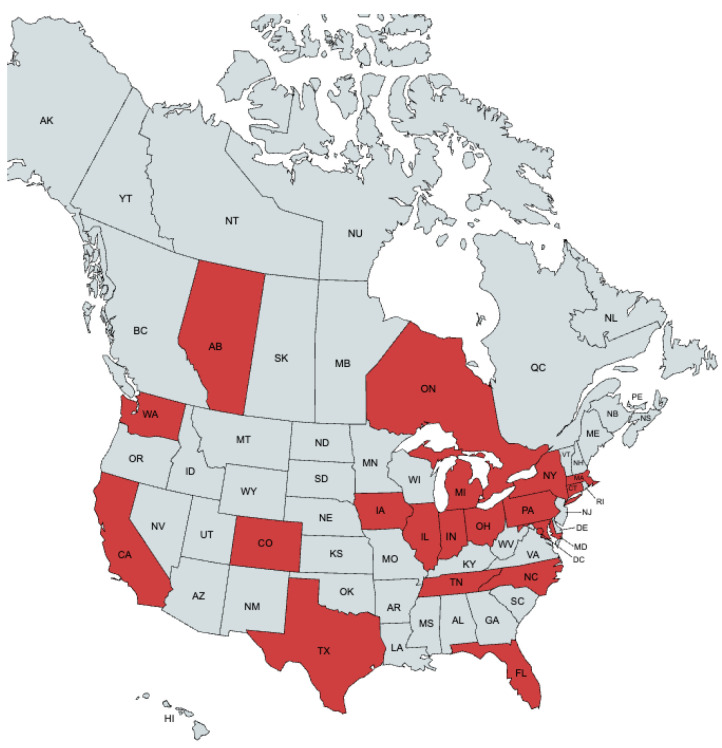
Geographic distribution of panelists. Red indicates the location of practice of a panelist.

**Figure 2:**
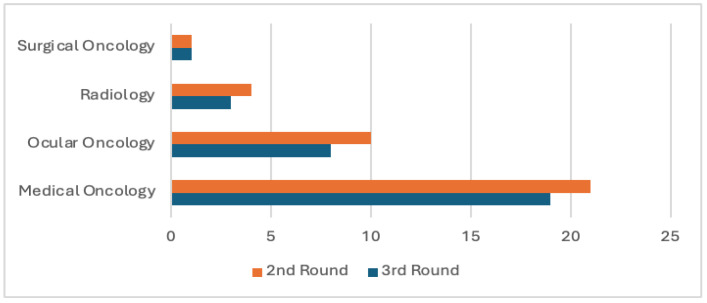
Specialty distribution.

Forty-nine experts from the United States and Canada were invited to participate in this Delphi study. A total of 41 experts from 17 U.S. states, Washington D.C., and 2 Canadian provinces completed the surveys (Figure 1). Consensus was achieved after three Delphi rounds. In rounds two and three, most participants were medical oncologists (21 and 19, respectively), followed by ocular oncologists (10 and 8), radiologists (4 and 3), and surgical oncologists (1 in each round) (Figure 2).

**Figure 3:**
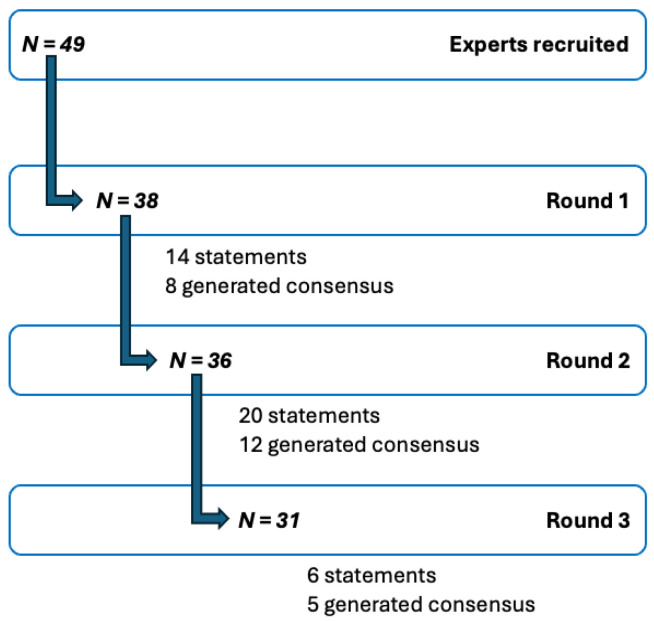
Modified Delphi process. Flow diagram of steps of the modified Delphi study, *N* = number of experts/panelists. A total of 41 panelists participated across three rounds of surveys.

In total, 41 panelists participated across the three surveys; not every panelist completed every survey. Response rates were 77% (38/49) in Round 1, 73% (36/49) in Round 2, and 63% (31/49) in Round 3 (Figure 3).

In Round 1, 14 statements were presented to the panelists, of which 8 achieved consensus. The remaining 6 statements were revised based on panelist feedback and reintroduced in Round 2. Additionally, 6 new statements were formulated, resulting in a total of 20 statements evaluated in Round 2. Of these, eight statements reaffirmed consensus, four achieved consensus for the first time, five failed to reach consensus for the first time, and three remained below the consensus threshold despite minor modifications based on feedback in the prior round. In Round 3, six statements were evaluated. Consensus was reaffirmed for three statements, one reached consensus for the first time, and one new statement did not reach consensus.

Two statements (#1 and #7) were almost identical to the current NCCN guidelines. Their inclusion in the modified Delphi allowed us to set a baseline consensus for our experts. Four statements regarding the high-risk group (#6–#9) and three others (#10–12) reached consensus in the first round and maintained consensus in the second round with minimal or no modifications. Three intermediate-risk group statements (#3–#5) reached consensus in the second and third rounds following significant modifications after panelist feedback. One intermediate-risk group statement (#2) did not meet consensus in the first round (66% agreement), but upon retesting in the third round, met consensus with a substantial increase in panelists in agreement (88%).

### 3.2. Final Consensus

#### 3.2.1. Intermediate-Risk Uveal Melanoma

1. Intermediate-risk patients should be screened for metastatic disease with a minimum frequency of every 3–6 months for 5 years, thereafter annually for at least 10 years from diagnosis.
2. In intermediate-risk patients, the use of contrast-enhanced cross-sectional imaging is preferred over hepatic ultrasound for surveillance of metastatic uveal melanoma.
3. In intermediate-risk patients, the use of contrast-enhanced MRI abdomen is preferred over hepatic ultrasound for surveillance of metastatic uveal melanoma.
4. In intermediate-risk patients, the use of contrast-enhanced MRI abdomen is preferred over CT abdomen for surveillance of metastatic uveal melanoma.
5. In intermediate-risk patients, a CT chest should be combined with hepatic imaging for surveillance of metastatic uveal melanoma; however, chest imaging may be performed at a reduced frequency.

#### 3.2.2. High-Risk Uveal Melanoma

6. High-risk patients should be screened for metastatic disease with a minimum frequency of every 3–6 months for years 1–5 and every 6–12 months for years 6–10. Surveillance imaging beyond 10 years may be considered.
7. In high-risk patients, the use of contrast-enhanced CT abdomen or MRI abdomen is preferred over hepatic ultrasound for surveillance of metastatic uveal melanoma.
8. In high-risk patients, the use of contrast-enhanced MRI abdomen is preferred over CT abdomen for surveillance of metastatic uveal melanoma.
9. In high-risk patients, a CT chest should be combined with hepatic imaging for surveillance of metastatic uveal melanoma; however, chest imaging may be performed at a reduced frequency.

#### 3.2.3. General Guidelines (Regardless of Risk Class)

10. Given the variability in FDG-avidity and the size threshold(s) required for PET/CT detection, PET/CT is not recommended for routine surveillance of uveal melanoma, regardless of risk class.
11. Patients with a history of hepatic steatosis or with an increased body habitus, which could lead to increased liver echogenicity or technical difficulties, should be screened with contrast-enhanced CT or MRI instead of hepatic ultrasound, regardless of their risk category.
12. Regardless of risk class, routine surveillance labs are unlikely to provide additional sensitivity or specificity to detect uveal melanoma metastases if imaging is obtained.

## 4. Discussion

This study used the modified Delphi method to create a consensus document regarding the surveillance of metastatic uveal melanoma in North America. We hope this consensus document will outline best practices for the surveillance of metastatic uveal melanoma. For both academic and community-based practitioners, some of whom may only occasionally encounter patients with UM, these expert consensus statements provide guidance regarding best practices for metastatic surveillance. This study is the first to incorporate both PRAME status and GEP into a comprehensive risk classification for metastatic UM. The risk classification was developed by the steering committee and integrates GEP, PRAME status, chromosomal alterations, tumor size, and mutational profile, reflecting the diverse criteria clinicians across North America use to determine metastatic risk.

Our expert panel, spanning across 16 US states, Washington D.C., and two Canadian provinces, provided a wide range of opinions, practice patterns, and site-based resources. To ensure our panelists had access to the most current literature on the topic during our study, we disseminated and discussed relevant publications from our literature review on surveillance in uveal melanoma during panel meetings. These studies were largely single-institution, retrospective in nature, and while relevant for understanding the depth of literature on the topic, their direct applicability was limited given their differing methodologies and the absence of survival data [10,12,21,22,23,24,25,26]. There was only one retrospective study with survival data in which the use of hepatic CT or MRI for surveillance of high-risk (Class 2 GEP) uveal melanoma patients was associated with the detection of smaller hepatic metastases compared to hepatic ultrasound; however, this did not translate to improved overall survival [24].

Despite regional differences in clinical practice, statements related to high-risk patients reached consensus in the first two rounds, indicating a shared degree of concern for the high likelihood of metastatic disease in this group. Additionally, statements #10–12, which focused on the use of PET/CT, routine labs, and the technical limitations of hepatic ultrasound, also reached immediate consensus. Our hope is that the inclusion of statements #10–12 will assist clinicians in better tailoring diagnostic testing for their specific patients’ needs. While statement #10 discourages the use of PET/CT for routine surveillance, the panel felt strongly that it can be considered in appropriate clinical circumstances as determined by the patient’s treating physician.

Reaching consensus on the optimal surveillance strategy for intermediate-risk patients was more challenging for the panel. These statements underwent significant modifications throughout all three rounds and occupied a large portion of our post-survey debriefing sessions. The debriefing sessions provided experts the opportunity to articulate their rationale for agreement or disagreement with each statement, thereby shaping the final recommendations listed above. This process was particularly impactful for statement #2, where agreement increased markedly from 66% in the first round to 88% in the third. Based on this rise, a fourth round for confirmation of consensus was deemed unnecessary. In the consensus statements, we emphasized that surveillance imaging of the abdomen with CT or MRI should be performed using contrast enhancement. However, recognizing institutional variations and differences in the availability of specific contrast agents such as Eovist, the panel chose not to prescribe a particular type of contrast, instead deferring this decision to the treating clinician’s discretion and per institutional guidelines. For chest imaging, although most panelists considered contrast unnecessary for routine surveillance, we acknowledged specific clinical scenarios where contrast might be beneficial, and thus we refrained from explicitly excluding its use. Ultimately, the most critical aspect of our consensus statements is that contrast-enhanced advanced hepatic imaging (CT or MRI) was favored over hepatic ultrasound in the intermediate- and high-risk settings.

On the other hand, in the low-risk group, despite wide agreement that different surveillance strategies are warranted compared to higher-risk groups, no consensus was reached as to the modalities, frequency, and duration of surveillance. The inability to achieve consensus on optimal surveillance strategies for low-risk patients was due in part to increasing recognition that the small percentage of low-risk UM patients who develop metastasis often do so much later in their disease course, compared to higher risk patients, and more frequently involve non-hepatic sites [4,14,27]. Furthermore, there was concern about potential risks associated with excessive surveillance in low-risk patients, including contrast and radiation exposure, false-positive results, additional biopsies, increased costs, and patient anxiety [4,23,28]. This challenge is further highlighted by a recent retrospective analysis of 144 low-risk (Class 1 GEP) UM patients, in whom metastatic disease occurred in only six patients (4%), and no significant differences in survival were found between surveillance modalities (CT/MRI versus hepatic ultrasound) [29]. The panel ultimately felt that insufficient data, rather than entrenched disagreement, was the primary barrier to consensus. Given the lack of consensus on the statements, we felt there would be diminishing returns in subsequent exploration of this topic in future rounds. As a result, no low-risk statements are included in the list of consensus statements. These findings underscore the complexity clinicians face in selecting appropriate surveillance strategies and reinforce the necessity for ongoing research to better inform clinical decision-making.

While our study provides clear recommendations for follow-up and imaging guidelines in the metastatic surveillance of uveal melanoma patients, we recognize the current lack of robust data to definitively demonstrate a survival advantage with more frequent or sensitive imaging. However, recent subgroup analysis from the phase 3 IMCgp100-202 trial highlighted that M1a uveal melanoma patients treated with Tebentafusp exhibited improved overall survival compared to those with more advanced disease (M1b/M1c) [30]. This finding suggests that the timely identification of metastatic disease could significantly enhance the therapeutic benefits of early intervention with this therapy.

A key consideration discussed during our panel debriefing sessions was the practical dilemma faced by medical and ocular oncologists when managing high-risk patients: while our guidelines advocate for frequent, sensitive imaging to detect metastatic lesions early, most medical oncologists would hesitate to move forward with liver-directed and/or systemic therapies in the absence of biopsy-proven disease. As a result, a substantial aspect of discussion in our debriefing sessions included the risk and benefit of identifying a hepatic lesion that is too small to biopsy. Ultimately, the panel felt that detecting a sub-centimeter lesion could serve as an early indicator of metastatic potential, warranting heightened vigilance. In such cases, increased surveillance using more sensitive and specific imaging modalities may be appropriate to facilitate earlier detection and intervention.

This study has several important limitations. First, the panel’s consensus was developed without high-quality prospective data. While the Delphi method is a well-established tool for obtaining structured expert input, the resulting statements reflect expert consensus rather than empirically validated guidelines. Although our primary focus was on clinical utility, the panel acknowledges the need for future studies to explore patient-centered outcomes and assess the economic feasibility of different surveillance strategies through cost-effectiveness analyses.

Of note, since the submission of our manuscript, an updated version of the UK Uveal Melanoma Guidelines [31] has been published, including recommendations that differ from ours, particularly regarding imaging modality and surveillance frequency. These differences are expected, as both sets of recommendations rely on expert consensus in the absence of high-quality prospective data, and variation naturally arises from regional practice patterns, available imaging resources, and preferred prognostic frameworks. Together, these efforts underscore the ongoing need for robust empirical evidence to better inform and harmonize surveillance strategies

Gene expression profiling (GEP) in uveal melanoma has practical limitations in routine clinical use, related primarily to cost, availability, and technical infrastructure requirements. The commercially available 15-GEP assay (DecisionDx-UM) is performed in a centralized laboratory and requires tumor material obtained via fine-needle aspiration biopsy (FNAB), along with specific specimen handling and shipment protocols, which may limit feasibility in some clinical settings [9,31]. In contrast, PRAME immunohistochemistry can be performed locally in most centers with standard pathology capabilities and represents a more accessible prognostic tool, although it provides complementary rather than equivalent prognostic information compared with GEP. In recognition of these considerations, contemporary guidelines incorporate PRAME status alongside GEP classification for risk stratification, supporting a more broadly applicable framework for prognostic assessment across diverse healthcare systems [9].

In conclusion, the modified Delphi survey technique identified consensus-driven recommendations for primary UM surveillance in intermediate- and high-risk patient populations. These guidelines have the potential to harmonize clinical practice patterns across the US and Canada. While these recommendations offer a valuable framework for clinicians, further work is needed to refine these guidelines based on empirical evidence, especially in low-risk patients. Future studies should prioritize prospective validation of surveillance strategies, assessing the impact of early metastatic detection on clinical outcomes and survival, the cost-effectiveness of surveillance, and the psychological burden of repeated imaging. Given the expanding therapeutic landscape, including immunotherapies, bispecific T-cell engager (BiTE) therapies, oncolytic viruses, cell therapy, targeted therapy, and immunomodulators in both the (neo)adjuvant and advanced settings, clear and standardized surveillance practices are critical to promptly identify patients who may benefit from these promising therapies. Lastly, we eagerly await the integration of emerging biomarkers and advanced imaging techniques that may further enhance surveillance precision and patient stratification, ultimately improving outcomes for patients diagnosed with UM.

## 5. Conclusions

This Delphi achieved consensus on surveillance imaging modalities, frequency, and duration for intermediate- and high-risk UM patients in North America. However, no consensus was reached on the optimal surveillance parameters for low-risk UM patients. Further research is needed to inform evidence-based guidelines. The consensus statements generated by this Delphi panel can provide a standardized framework to improve clinical practice and surveillance consistency for patients with UM. Importantly, these recommendations are based on expert opinion rather than prospective evidence and should be interpreted as provisional guidance pending empirical validation.

## Data Availability

The raw data supporting the conclusions of this article will be made available by the authors on request.

## Abbreviations

The following abbreviations are used in this manuscript:

UM	Uveal melanoma
MUM	Metastatic uveal melanoma
GEP	Gene-expression profile
COOG	Collaborative Ocular Oncology Group
PRAME	Preferentially expressed antigen in melanoma
MFS	Metastasis-free survival
NCCN	National Comprehensive Cancer Network
AJCC	American Joint Committee on Cancer
